# Factors Associated With the Presence and Severity of Nutritional Impact Symptoms in Individuals With Head and Neck Cancer Before Treatment

**DOI:** 10.1155/ijso/3390646

**Published:** 2024-12-28

**Authors:** Thainá C. do Rosario, Fabíola L. P. Soares, Louise V. O. Soares, Julia S. N. Gallavotti, Isabela S. Rodrigues, Camila B. do Prado, Olívia P. G. de Podestá, Katia Cirlene G. Viana, Ricardo M. Rocha, Jeferson Lenzi, José Roberto V. de Podestá, Evandro D. de Souza, Fabiano K. Haraguchi, Glenda B. Petarli, André S. Leopoldo, Luciane B. Salaroli

**Affiliations:** ^1^Federal University of Espírito Santo (UFES), Vitória, Espírito Santo, Brazil; ^2^Faculdade Multivix, Vitória, Espírito Santo, Brazil; ^3^Hospital Santa Rita de Cássia/Afecc, Vitória, Espírito Santo, Brazil; ^4^Clinical Nutrition Unit, Cassiano Antonio Moraes Hospital (HUCAM), Vitória, Espírito Santo, Brazil

**Keywords:** head and neck neoplasms, nutritional status, risk, signs and symptoms

## Abstract

**Background:** As head and neck cancer (HNC) affects regions directly related to the digestive tract, it is consistently associated with nutritional impact symptoms (NISs), which further reduce food intake and affect nutritional status. Early identification of patients with NIS can assist therapy.

**Method:** This is a cross-sectional study with HNC patients from a cancer reference hospital. Sociodemographic, lifestyle, clinical, and anthropometric data were collected, along with information on nutritional risk screening and screening for NIS.

**Results:** Cancer in the larynx (*p*=0.031) showed a 6.67 lower NIS score than that in the oral cavity. Ex-smokers (*p*=0.019) showed a 5.87 lower NIS score and nutritional risk (*p*=0.009) increased NIS scores by 6.15 points.

**Conclusion:** Tumor location, smoking, and the presence of nutritional risk influence the quantity and severity of NIS.

## 1. Introduction

Head and neck cancer (HNC) refers to a group of heterogeneous tumors that originate, except for skin tumors, in the mucosa of the upper gastrointestinal tract, especially in the oral cavity, pharynx, and larynx [1]. It ranks seventh in incidence and eighth in mortality estimates among the most prevalent cancers worldwide, excluding nonmelanoma skin cancer [1, 2].

HNC is a multifactorial disease whose risk factors arise from the interaction between environmental influences and genetic inheritance [3]. Among environmental factors, smoking has emerged as the greatest risk factor, followed by alcohol consumption. When consumed simultaneously, risk increases from 10 to 100 times [4–6].

As HNC affects regions directly related to the digestive tract (reducing food intake) and induces a hypercatabolic state, carriers show significant weight loss from the time of diagnosis [7, 8]. Such nutritional decline is consistently associated with nutritional impact symptoms (NISs). NISs are physiological alterations caused by cancer that can compromise oral intake, including pain, dry mouth, difficulty swallowing, reduced appetite, and vomiting, among others [9, 10]. Approximately 96% of the population with HNC show one or more NISs before starting treatment [11].

Individuals with HNC and multiple NISs are more likely to experience reduced food intake, weight loss, functional capacity dysfunction, and decreased survival [12–14]. Systematic assessment and management of these symptoms before the start of treatment are crucial to prevent serious nutritional complications that often develop during antineoplastic therapy [13]. An early approach allows for the implementation of appropriate nutritional and therapeutic strategies that can not only mitigate the progression of symptoms but also significantly improve the patients' quality of life. Furthermore, early treatment of NIS can prevent patients from reaching a state of severe malnutrition, which would further compromise the effectiveness of the treatment and survival [13, 14].

Therefore, early recognition (pretreatment) of patients with a higher burden of NIS is crucial and provides insights that can assist in appropriate therapy before the development of malnutrition and associated factors. This study aimed to analyze the presence and severity of NISs and their associated factors in individuals with HNC before treatment.

## 2. Materials and Methods

### 2.1. Methodological Design and Study Population

This is an epidemiological, analytical, and cross-sectional investigation of individuals with HNC who were treated at a reference oncology hospital in Greater Vitória, Espírito Santo, Brazil. The data represent the baseline of a longitudinal study titled “Nutritional Indicators, Mortality, and Associated Factors: A Hospital-Based Study in Individuals with HNC.” The target population consisted of patients diagnosed with squamous cell carcinoma (mouth, larynx, oropharynx, and hypopharynx) of all sexes who were aged 18 years and above.

### 2.2. Sampling and Selection

The study participants were invited to join after confirmation of their HNC diagnosis by the Head and Neck Surgery Medical Service at a cancer treatment reference hospital. The participants were patients receiving care through the Unified Health System (SUS), Brazil's public healthcare system. Following histopathological confirmation of the HNC diagnosis, all eligible individuals were invited by the researchers between September 2022 and January 2024. Participation was confirmed by signing informed consent forms that detailed the research. Data were collected by researchers who had undergone prior training in administering questionnaires and performing anthropometric measurements, including the standardization of measurement techniques to ensure data accuracy.

To calculate the sample size, a sample universe of 200 patients was considered, corresponding to the average annual number of first consultation visits at the HNC outpatient clinic, a prevalence of 50% (to maximize the sample), a sampling error of 5%, and a confidence interval of 95%. The resulting minimum sample size totaled 132 individuals. The calculation was performed using Epi Info 7.2 software.

### 2.3. Inclusion and Exclusion Criteria

Participants of all sexes who were aged 18 years or above, diagnosed with squamous cell carcinoma of the oral cavity, larynx, oropharynx, and hypopharynx, and without previous treatment, were included. Cases were confirmed by histology and classified according to the ICD-10-03 [15] into the following topographies: base of tongue (C01), other and unspecified parts of tongue (C02), gum (C03), floor of mouth (C04), palate (C05), other and unspecified parts of mouth (C06), tonsil (C09), oropharynx (C10), pyriform sinus (C12), hypopharynx (C13), and larynx (C32).

Subsequently, the ICD codes were grouped into four topographic categories: oral cavity (C01, C02, C03, C04, and C05), oropharynx (C05.1, C09, and C10), larynx (C32), and hypopharynx (C12 and C13). Patients were excluded from the study if they had a diagnosis of recurrent squamous cell carcinoma, had previously received cancer treatment, presented with multiple tumors, or lacked the clinical and mental capacity to respond to the administered questionnaire.

### 2.4. Data Collection

A questionnaire was administered to evaluate patients' sociodemographic (sex, age, race/ethnicity, education, and income), lifestyle (smoking and alcohol consumption), and clinical data (tumor site and staging). It is worth noting that sociodemographic variables and lifestyle habits were self-reported. The Head and Neck Symptom Checklist (HNSC), a validated tool in individuals with HNC [10], was used to assess NIS. It consists of 17 symptoms assessed on a five-point Likert scale ranging from “1, not at all” to “5, very much.” For analysis, all 17 symptom scores were added, resulting in a total score that can range from 17 (no symptoms) to 85 (highest score for each symptom on the list) [10].

The Nutritional Risk Screening (NRS-2002) tool was used to assess nutritional risk. It is a validated instrument [16] and is based on data on dietary intake in the previous week, body mass index (BMI), weight loss in the last three months, age, and disease severity. Patients are scored according to malnutrition and the severity of the underlying disease, classified for each variable as absent (0), mild (1), or severe (2). Patients with a score of three or more are classified as at nutritional risk, while those with a score below three should be reassessed [16]. In addition, anthropometric measurements (weight and height) were collected by the researchers, and body composition measurements (appendicular skeletal muscle mass) were obtained using a portable InBody 120 bioimpedance device.

### 2.5. Statistical Analysis

Data were evaluated on Research Electronic Data Capture [17, 18], uploaded to the cloud of the Federal University of Espírito Santo, and organized and analyzed on R (4.3.1) for Windows. Absolute and relative frequencies were used to describe the categorical variables. Measures of central tendency (means and medians) and dispersion (standard deviations and interquartile ranges) were used to describe continuous variables. NIS scores (17–85 points) were used as the dependent variable. A multiple linear regression was performed to quantify the participation of independent variables (sex, age, staging, education, income, topography, race/color, smoking, alcohol consumption, nutritional risk, BMI, and ASMM) in the outcome. The significance level for all tests was set at 5%.

### 2.6. Ethical Aspects

This project was approved by the Research Ethics Committee of the Cassiano Antônio Moraes University Hospital under final opinion no. 6,241,277 and CAAE 56453322. 7.0000.5060. It complied with the ethical standards governing research involving human subjects.

## 3. Results

The sample consisted of 132 participants, with a higher proportion of older adults (61%)—with a mean age of 61 years, men (77%), mixed-race people (54%), with less than eight years of education (71%), and a family income of less than or equal to two minimum wages (76%). In terms of lifestyle habits, most participants reported being former smokers (52%) and former drinkers (65%). However, even after diagnosis, 19% continued to consume alcohol. The most prevalent topography was in the oral cavity (45%), with stage IV tumors (53%). Although 43% of participants were overweight (showing overweight and obesity), 45% of the population were at nutritional risk according to the NRS at the time of diagnosis (Table 1).

Regarding NIS, 95% of participants showed one or more NISs (*n* = 126), with the most recurrent symptoms being pain (68.93%), difficulty chewing (60.60%), thick saliva (59.84%), anxiety (58.33%), sore mouth (52.27%), and difficulty swallowing (50.0%) (Table 2).

Table 3 shows the data on the association between sociodemographic, lifestyle, and clinical variables and the total score of NISs. Topography, smoking, and nutritional risk showed an association. Cancer in the larynx (*p*=0.031) had an estimated reduction of 6.67 points in NIS scores in comparison to that in the oral cavity. Regarding smoking, former smokers (*p*=0.019) showed an estimated reduction of 5.87 points in NIS scores than smokers. Finally, participants with nutritional risk (*p*=0.009) have an estimated increase of 6.15 points in total NIS scores when compared to those without nutritional risks (see Table 4).

## 4. Discussion

This study evinced a high severity and quantity of NIS in its population. About 9 out of 10 participants reported one or more NISs before treatment, corroborating the findings by Farhangfar et al. [13], who observed that 94% of their HNC cohort had one or more NISs before antineoplastic therapy. NISs lead to a series of consequences for individuals, inducing an inflammatory response that limits energy and protein intake and contributes to increased stress and weight loss before and even during treatment [8, 9]. Additionally, NIS presence and severity tend to increase during treatment [19], which can impact treatment efficacy and sometimes necessitate treatment interruption until the specific symptom is reversed and the individual can resume therapy. Jin et al. [19] showed that NIS worsened during radiotherapy early on, emphasizing the importance of managing these symptoms before starting therapy.

The most recurrent symptoms included pain (68.93%), difficulty chewing (60.60%), thick saliva (59.84%), anxiety (58.33%), sore mouth (52.27%), and difficulty swallowing (dysphagia) (50.0%). Results showed symptom clusters similar to previous studies. Kubrak, Olson, and Baracos [10] found that the most common symptoms before treatment were pain (33%) and dysphagia (29%). Farhangfar et al. [13] reported that the most prevalent symptoms were pain (63.6%), anxiety (62.9%), and lack of energy (58.5%). Furthermore, Granstrom et al. [11] showed that the highest NIS scores at baseline occurred for pain (96%), anxiety (96%), and sore mouth (95%). Despite the discrepancies in symptoms across studies, pain is commonly the most reported symptom, typically ranking first in proportions. This study may explain this fact by the predominant location (the oral cavity) and the advanced stage of the disease (stage IV).

This study found an association between tumor location and NIS score before treatment. Individuals with HNC in the larynx had NIS lower quantity and/or severity than those with cancer in their oral cavity. These findings resemble those by Granstrom et al. [11], who found a higher NIS score in individuals with HNC in the oral cavity and oropharynx than in those with larynx cancer during and after treatment. The main signs and symptoms in laryngeal cancer include hoarseness, pain during swallowing, changes in voice quality, sensation of lumps in the throat, difficulty breathing, or even shortness of breath [20]. Of these reported symptoms, the HNSC—the tool chosen for the study—includes only one: odynophagia (painful swallowing), with only 12% of the population reporting this type of NIS. Therefore, a partial explanation for a lower presence and/or severity of symptoms in laryngeal cancer may be due to the chosen tool ignoring the other characteristic symptoms of this type of cancer.

Individuals who quit smoking had lower NIS presence and/or severity than those who still smoked. Smoking is a well-established direct risk factor in the literature, considered the primary risk factor in HNC development [4, 21]. Tobacco acts as a factor for tumor growth, and cigarette smoke acts as a mutagenic and DNA-damaging agent that drives tumor initiation in normal epithelial cells [21, 22]. Thus, individuals who continue smoking after diagnosis contribute to this entire cascade driving the tumor, which is consistently related to a higher burden or severity of NIS.

The presence of nutritional risk in individuals with HNC is relatively high from the time of diagnosis. Overall, 45% of the population in this study was at nutritional risk, agreeing with the findings by Wang et al. [23] and by Bossi et al. [24], who found around 30.3% of the HNC population showing nutritional risk at the time of diagnosis. Furthermore, the presence of nutritional risk and total NIS scores showed an association. Individuals at nutritional risk had a higher presence or severity of NIS than those without nutritional risk. According to Wang et al. [23], NISs are positively correlated with nutritional risk in HNC patients in the perioperative period, resulting in higher scores according to the NRS-2002.

As a possibility of bias, information bias was minimized due to training of all staff in data collection and management. In addition, selection bias was also a possibility; however, all individuals who were in the CCP outpatient clinic during the research period were invited to participate in the research. The refusal rate was low, at 2.1%, out of a total of 143 individuals.

This study has limitations. Its cross-sectional design implies an analysis of NIS at a single point in time, which affects causality. Additionally, a multicenter evaluation with a larger number of patients could provide more accurate assumptions. However, the findings in this study complement the literature and show that health education and nutritional monitoring should be provided to patients from the moment of diagnosis to help them understand the importance of nutritional treatment, ensuring adequate nutritional intake and improving their nutritional status.

## 5. Conclusion

Individuals with HNC who smoke and have nutritional risk show higher estimates of NIS severity and quantity. However, those with tumors in their larynx have lower estimates of symptom severity than those with cancer in their oral cavity.

## Figures and Tables

**Table 1:** List of 17 symptoms of nutritional impact contained in Head and Neck Symptom Checklist (HNSC).

Nutritional impact symptoms (NISs)	Classification by Likert scale
Pain	1 (not at all) to 5 (very much)
Anxiety	1 (not at all) to 5 (very much)
Feeling of fullness	1 (not at all) to 5 (very much)
Constipation	1 (not at all) to 5 (very much)
Loss of appetite	1 (not at all) to 5 (very much)
Dry mouth	1 (not at all) to 5 (very much)
Depression	1 (not at all) to 5 (very much)
Thick saliva	1 (not at all) to 5 (very much)
Diarrhea	1 (not at all) to 5 (very much)
Sore mouth	1 (not at all) to 5 (very much)
Lack of energy	1 (not at all) to 5 (very much)
Nausea	1 (not at all) to 5 (very much)
Difficulty chewing	1 (not at all) to 5 (very much)
Altered smell	1 (not at all) to 5 (very much)
Vomiting	1 (not at all) to 5 (very much)
Difficulty swallowing	1 (not at all) to 5 (very much)
Altered taste	1 (not at all) to 5 (very much)

**Table 2:** Sociodemographic, lifestyle, and clinical characteristics of 132 patients with head and neck cancer, Vitória, 2022–2024.

Variables		*N* (%)
Sex	Female	30 (23)
	Male	102 (77)

Age (years)		61 (±12)⁣^∗^

Age	Adult	52 (39)
	Older adult	80 (61)

Education (years)	< 8	94 (71)
	≥ 8 to 11	23 (17)
	≥ 11	15 (11)

Race/ethnicity	White	43 (33)
	Brown	71 (54)
	Black	17 (13)
	Indigenous	1 (0.8)

Income	≤ 2 MW	99 (76)
	> 2 MW	33 (25)

Topography	Oral cavity	59 (45)
	Hypopharynx	13 (9.8)
	Larynx	21 (16)
	Oropharynx	39 (30)

Staging, UICC 8	I	7 (5.3)
	II	27 (21)
	III	28 (21)
	IV	69 (53)

Smoking	Yes	38 (29)
	Former smoker	69 (52)
	Never	25 (19)
	Pack-years of smoking	130 (54–360)^†^

Alcohol consumption	Yes	25 (19)
	Former drinker	86 (65)
	Never	21 (16)

Nutritional risk	At risk	60 (45)
	No risk	72 (55)

Muscle mass		7.05 (±1.24)

BMI (kg/m^2^)		23.9 (±5.6)⁣^∗^

BMI	Underweight	27 (20)
	Normal weight	48 (36)
	Overweight	40 (30)
	Obesity	17 (13)

Abbreviations: BMI = body mass index, MW = minimum wage, and UICC 8 = Union for International Cancer Control eighth edition.

⁣^∗^Mean (±SD).

^†^Median (interquartile range).

**Table 3:** Presence and severity of NIS.

Nutritional impact symptoms	*N* (%)	Median (±DP)
Pain	91 (68.93)	2.9 (±1.6)
Anxiety	77 (58.33)	2.6 (±1.6)
Feeling of fullness	38 (28.78)	1.8 (±1.4)
Constipation	29 (21.96)	1.6 (±1.3)
Loss of appetite	49 (37.12)	1.9 (±1.3)
Dry mouth	51 (38.63)	2.1 (±1.5)
Depression	51 (38.63)	2.0 (±1.5)
Thick saliva	79 (59.84)	2.8 (±1.7)
Diarrhea	06 (4.54)	1.1 (±0.4)
Sore mouth	69 (52.27)	2.5 (±1.7)
Lack of energy	51 (38.63)	2.0 (±1.5)
Nausea	21 (15.90)	1.3 (±0.9)
Difficulty chewing	80 (60.60)	3.0 (±1.8)
Altered smell	27 (20.45)	1.6 (±1.3)
Vomiting	08 (6.06)	1.1 (±0.5)
Difficulty swallowing	66 (50.00)	2.5 (±1.7)
Altered taste	29 (21.96)	1.7 (±1.4)
Total score	126 (95.00)	34.4 (±11.3)

*Note: N* = 132; mean and standard deviation values for symptom severity (1–5).

**Table 4:** Association between sociodemographic, lifestyle, and clinical variables and the total score of NIS severity according to the HNSC.

Variables		Estimate	CI_95%_		*p* value
			Bottom	Higher	
NIS	Intercept	41.895	−21.37	26.72	**< 0.001**

Sex⁣^∗^	Female	4.99	−0.85	11	0.093

Age		−0.06	−0.25	0.12	0.480

Staging^†^	I	−5.16	−14	3.6	0.244
	II	3.62	−1.9	9.0	0.197
	III	0.02	−5.2	5.3	0.992

Education^‡^	≥ 8–11 years	−0.40	−5.6	4.7	0.876
	≥ 11 years	−0.44	−7.0	6.1	0.891

Topography^§^	Hypopharynx	2.44	−4.4	9.3	0.483
	Larynx	−6.67	−13	−0.61	**0.031**
	Oropharynx	1.24	−4.2	5.6	0.608

Race/color⁣^‖∡^	White	−2.13	−6.6	2.3	0.345
	Black	−0.74	−6.8	5.4	0.810

Smoking^ ¶^	Ex-smoker	−5.87	−11	−0.98	**0.019**
	Never	−6.05	−14	1.7	0.123

Alcohol consumption^#^	Former drinker	3.57	−2.2	9.1	0.204
	Never	2.69	−5.1	10	0.492

Nutritional risk^¥^	Nutritional risk	6.15	1.1	11	**0.009**

BMI		−0.11	−0.68	0.33	0.639

ASMM^$^	Low ASMM	0.73	−3.5	5.0	0.732

*Note: N* = 132. The association was estimated by multiple linear regression. Estimates were interpreted as real increases in the sums of NIS scores (range from 17 to 85). Values in bold indicate statistically significant results.

Abbreviations: ASMM = appendicular skeletal muscle mass; BMI = body mass index.

⁣^∡^*N* = 131.

⁣^∗^Reference: male.

^†^Reference: IV.

^‡^Reference: < 8 years.

^§^Reference: oral cavity.

^‖^Reference: brown.

^¶^Reference: smoker.

^#^Reference: drinker.

^¥^Reference: no risk.

^$^Reference: preserved appendicular skeletal muscle mass.

## Data Availability

The data that support the findings of this study are available on request from the corresponding author. The data are not publicly available due to privacy or ethical restrictions.
